# Excretory/secretory proteins inhibit host immune responses by downregulating the TLR4/NF-κB/MAPKs signaling pathway: A possible mechanism of immune evasion in parasitic nematode *Haemonchus contortus*

**DOI:** 10.3389/fimmu.2022.1013159

**Published:** 2022-09-27

**Authors:** Zhaohai Wen, Yue Zhang, Jiajun Feng, Kalibixiati Aimulajiang, Muhammad Tahir Aleem, Mingmin Lu, Lixin Xu, Xiaokai Song, Xiangrui Li, Ruofeng Yan

**Affiliations:** ^1^ Ministry of Education Joint International Research Laboratory of Animal Health and Food Safety, College of Veterinary Medicine, Nanjing Agricultural University, Nanjing, China; ^2^ State Key Laboratory of Pathogenesis, Prevention and Treatment of High Incidence Diseases in Central Asia, Clinical Medicine Institute, The First Affiliated Hospital of Xinjiang Medical University, Urumqi, China

**Keywords:** *Haemonchus contortus*, HcESPs, PBMCs, TLR4/NF-κB/MAPKs signaling pathway, immune evasion, pattern recognition receptor

## Abstract

*Haemonchus contortus* is an important parasitic nematode of ruminants. Previous studies showed that *H. contortus* escape the immunity through complex mechanisms, including releasing excretory/secretory proteins (ESPs) to modulate the host immune response. However, the detailed mechanism through which *H. contortus* excretory/secretory proteins (HcESPs) promote immune evasion remains unknown. In the present study, we demonstrated that HcESPs inhibit the adaptive immune response of goats including downregulation of immune cell antigen presentation, upregulation of immune checkpoint molecules, activation of the STAT3/PD-L1 pathway, and activation of immunosuppressive regulatory T (Treg) cells. Furthermore, HcESPs reversed the LPS-induced upregulation of pro-inflammatory mediators in PBMCs by inhibiting the TLR4/NF-κB/MAPKs/NLRP3 signaling pathway. Our study provides a better understanding of the evasion mechanisms for *H. contortus*, which could be helpful in providing an alternative way to prevent the infection of this parasite.

## Introduction

Parasites are grossly neglected pathogens that infect humans, animals, and crops (1–4). Particularly, parasitic nematodes cause severe morbidity and mortality worldwide (1–4). *Haemonchus contortus* is one of the most distributed parasites for ruminants, causing enormous losses to the global livestock industry and negatively impacting animal welfare (5). Unlike viruses and bacteria, parasitic nematodes establish a long-term infection in the host, usually with only a mild inflammatory response. This is attributed to the release of complex excretory/secretory protein (ESP) mixtures into host tissues that interfere with host signaling mechanisms and immune homeostasis (6). However, the detailed mechanism of the role of nematode ESP in immune evasion remains to be further investigated.

Previous studies in our laboratory found that the *H. contortus* ESPs (HcESPs) inhibited the proliferation and nitric oxide (NO) release from PBMCs *in vitro* (7). However, we know little about the molecular mechanisms, including the related signaling pathways that HcESPs employ to suppress the host innate and adaptive immunity.

Immune checkpoints are inhibitory pathways in the immune system, regulated by the interaction of receptors expressed on immune cells with their ligands (8). LAG-3 (Lymphocyte-activation gene 3), PD-1 (programmed death 1), PD-L1 (programmed death ligand 1), and CTLA-4 (cytotoxic lymphocyte-associated antigen 4) are immune checkpoint molecules that cause immune cell dysfunction and are negative regulators of the immune response (9, 10). Studies have shown that many pathogens (including parasites, bacteria, and viruses) can use the PD-1 pathway to evade host adaptive immunity (11–14). During *Trypanosoma cruzi* infection, blockade of CTLA-4 resulted in increased host resistance to *T. cruzi* infection, which was associated with increased production of IFN-γ, TNF-α, and NO (15). Butler NS et al. reported that therapeutic blockade of LAG-3 and PD-L1 rapidly cleared blood-stage *Plasmodium vivax* in mice (16). However, the role of immune checkpoint molecules in *H. contortus* infection has not been reported.

Pattern recognition receptor (PRR) recognition of pathogen-associated molecular patterns (PAMP) is critical in initiating innate immune responses in animals. The immune system can detect invading pathogens through toll-like receptors (TLRs) and instruct the subsequent immune response to clear the infection (17). TLRs recognize PAMPs of various pathogens, such as parasites, bacteria, viruses, and fungi. TLRs initiate complex signaling cascades within cells, such as promoting the release of inflammatory mediators, promoting the formation of inflammasomes, and thus activating various innate immune responses. The innate immune response is the host’s first line of defense against pathogen invasion, and TLRs are a vital component of the immune defense; therefore, many pathogens have evolved strategies to manipulate the TLRs signaling pathway to suppress the host response (16, 18–22). However, nothing is known about the effect of HcESPs on goat PRR.

In this study, we analyzed the effects of HcESPs on goats in antigen presentation, expression of immune checkpoint molecules, expression of Tregs cell marker molecules, and STAT3/PD-L1 signaling pathway in PBMCs to investigate the molecular mechanisms by which HcESPs regulate adaptive immune responses. Moreover, we also analyzed the effect of HcESPs on the transcriptional levels of PRR genes in PBMCs. Furthermore, we analyzed the anti-inflammatory effects of different concentrations of HcESPs and its impact on inflammatory pathways such as nuclear factor-kappa B (NF-κB) and Mitogen-activated protein kinases (MAPKs) by establishing an inflammation model of PBMCs using lipopolysaccharides (LPS). Our study identified the molecular mechanisms involved in the modulation of immune cell function caused by HcESPs and the associated signaling pathways, which provides new insights to understand the strategies employed by *H. contortus* to survive in the host.

## Methods

### Parasites and animals

The 3- to 6-month-old local goats were housed in the experimental animal center of the college of veterinary medicine, Nanjing Agricultural University, and provided with sterile hay, corn feed, and free access to water. *H. contortus* (Nanjing strain) were maintained by successive passages of goats *in vivo*.

### Collection of HcESPs and isolation of goat PBMCs

Adult *H. contortus* (females and males) and HcESPs were collected as described in previous studies (7, 23). Briefly, each goat was orally infected with 8000 infective third-stage larvae (iL3s). The excretion of fecal egg was checked since 21 days post infection (DPI), and adult worms were collected from the fourth stomach of goats at 35 DPI. About 300 to 500 worms were collected from each goat. The worms were washed with sterile phosphate-buffered solution (PBS), and cultured in RPMI 1640 medium (100 worms/mL) containing 1% penicillin and 1% streptomycin at 37°C in a 5% CO_2_ incubator for 24 h. HcESPs in the culture supernatant was collected, desalted, and concentrated using ultrafiltration tubes (3 kDa-cut-off). The concentration of HcESPs was determined by the BCA method and confirmed by SDS-PAGE (**Supplementary material Figure S1**). Total of 48 mg of HcESPs were collected by incubation of 400 adult worms for 24 h. The possible concentration of HcESPs in infected goat was estimated from 34 to 86 µg/mL. However, differences in host body weight and worm burden might affect the assessment of physiological concentrations of HcESPs.

Goat PBMCs were isolated from peripheral blood using the standard Ficoll-Hypaque (GE Healthcare, Little Chalfont, UK) gradient centrifugation method described previously (24). PBMCs were washed three times with PBS, and the cell density was adjusted to 1 × 10^6^ cells/mL. Then, the cell viability was detected with Trypan blue dye (25).

### Cell proliferation assays

Cell proliferation was assayed with the Cell Counting Kit-8 (CCK-8, Beyotime) with reference to previous studies (26). Briefly, freshly isolated PBMCs (concentration of 1×10^6^/mL) were homogenously added to 96-well cell culture plates at 100 μL per well. Subsequently, HcESPs at different concentrations (10, 20, 40, 80 μg/mL) were added to co-incubate with PBMCs, and a control group was established (equal volume of RPMI 1640 medium instead of HcESP). The cell culture plates were incubated in a 5% CO_2_ cell incubator at 37°C for 24 h. Then, 10 μL of CCK-8 solution was added to each well, and incubated for another 2 h, the absorbance values at OD_450_ were measured by a microplate reader.

### Transcriptional abundance of macrophage marker molecules, MHC class II molecules, immune checkpoint molecules, Treg cell marker molecules, and PRRs in PBMCs were analyzed by qPCR assays

PBMCs (concentration of 1×10^6^/mL) were homogenously added to 6-well cell culture plates at 2 mL per well. Different concentrations (5, 10, 20, 40, 80 μg/mL) of HcESPs were added and co-incubated with PBMCs, and a control group was established by adding an equal volume of RPMI 1640 medium. The cells were incubated for 0, 6, 12, 18, and 24 h at 37°C in a 5% CO_2_ incubator. Subsequently, the cells were collected for RNA extraction.

Total RNA was extracted from PBMCs by the TRIzol method (Vazyme Biotech, Nanjing, Jiangsu, China) according to the manufacturer’s protocol. The cDNA was synthesized by the HiScript III 1st Strand cDNA Synthesis Kit (Vazyme Biotech, Nanjing, Jiangsu, China). The reaction system contains 3.4 μL ddH_2_O, 0.2 μL forward and reverses primers, 5 μL 2×ChamQ SYBR qPCR Master Mix (Vazyme Biotech co.,ltd), and 1 μL cDNA. The qPCR reaction was performed under the following conditions: one cycle at 95°C/30 s; 40 cycles at 95°C/10 s, 60°C/30 s; and one cycle at 95°C/15 s, 60°C/60 s, and 95°C/15 s. The β-actin was used as an internal reference gene (27). Primer sequences for Arg1, iNOs, CLA-DQB1, CLA-DQB3, PD-L1, PD-1, LAG-3, CTLA-4, IL-10, TGF-β, Foxp3, and PRRs were shown in the **Supplementary material Table S1**. The data were analyzed using the comparative Ct (2^−ΔΔ Ct^) method, based on cycle thresholds (Ct), which were obtained by ABI Prism 7500 software (Applied Biosystems, Foster City, CA, USA).

### Antigen uptake by PBMCs

The effect of HcESPs on the antigen uptake of PBMCs was determined by detecting FITC-dextran internalization. FITC-dextran was used as a fluorescent antigen, and FITC fluorescence was observed in PBMCs after phagocytosis of FITC-dextran. Therefore, the phagocytic function of PBMCs can be assessed by the fluorescence intensity. Briefly, different concentrations (10, 20, 40, 80 μg/mL) of HcESPs were co-incubated with PBMCs for 24 h at 37°C in a 5% CO_2_ incubator, and a control group was established by adding an equal volume of RPMI 1640 medium. The PBMCs were then incubated with 100 ng/mL FITC-dextran for 1 h. The cells were subsequently washed 3 times with PBS and placed under a fluorescent microscope for observation. The phagocytosis was assessed by the intensity of FITC-dextran in PBMCs.

### Expression of key molecules of Treg cells and STAT3/PD-L1 pathway in PBMCs was analyzed by western blot assays

Freshly isolated PBMCs (concentration of 1×10^6^/mL) were homogenously added to 6-well cell culture plates at 2 mL per well. Subsequently, HcESPs at different concentrations (10, 20, 40, 80 μg/mL) were added and co-incubated with PBMCs, and a control group was established by adding an equal volume of RPMI 1640 medium. The cells were incubated for 24 h at 37°C in a 5% CO_2_ incubator. Subsequently, the cells were collected for total protein extraction.

Western blot assays were performed with reference to the methods of previous studies (28). Briefly, total protein was obtained by lysing PBMCs using RIPA Lysis Buffer (Beyotime, Shanghai, China), and the concentration of protein samples was determined by the BCA kit (Beyotime, Shanghai, China). The proteins (30µg) were transferred onto PVDF membranes (GE Healthcare Life Science, Beijing) by 10% SDS-PAGE. To block the non-specific binding sites, the membranes were incubated with 5% BSA for 2h at room temperature. The membranes were washed 5 times with TBST and incubated with primary antibody at 4°C overnight. Washed with TBST, the membranes were incubated with goat anti-rabbit IgG (H+L) HRP secondary antibody for 2 h at room temperature. The expression of β-actin was used as an internal quantitative control. The expression of the target proteins was detected by chemiluminescence. Image J was used to analyze the relative protein expression levels.

Source antibodies: TGF-β1 (1:500; Santa Cruz Biotechnology, Inc., Dallas, TX, USA); IL-10, PD-L1 p705-STAT3 (1:1000; Affinity Biosciences, Jiangsu province, China); β-actin (ABclonal, China).

### Inflammation modeling

To investigate the effect of HcESPs on inflammation and inflammation-related signaling pathways, we used Lipopolysaccharides (LPS) to treat PBMCs to establish an inflammation model. Briefly, freshly isolated PBMCs (concentration of 1×10^6^/mL) were homogenously added to 6-well cell culture plates at 2 mL per well. Then, the PBMCs were stimulated using LPS (1 µg/mL), and series concentrations (0, 10, 20, 40, 80 μg/mL) of HcESPs were co-incubated with PBMCs for 24 h at 37°C in a 5% CO_2_ incubator. At the same time, a blank control group (RPMI 1640 medium instead of LPS and HcESP) was set up.

### Transcriptional abundance of cytokines and related pathway molecules detected by qPCR assays

Group settings were consistent with the description in section 2.7 (“Inflammation modeling”). The detailed procedure for qPCR assays is consistent with the description in Section 2.3. The primers TNF-α, IL-6, IL-1β, TLR4, myD88, ASC, NLRP3, and IL-18 for qPCR are listed in **Supplementary material Table S1**.

### The expression abundance of cytokines and related pathway molecules detection by western blot assays

Group settings were consistent with the description in section 2.7 (“Inflammation modeling”). The detailed procedure for western blot assays is consistent with the description in Section 2.5. Source antibodies: IL-1β, TNF-α, TGF-β1, TLR4 (1:500; Santa Cruz Biotechnology, Inc., Dallas, TX, USA); IL-6, IL-10, p705-STAT3, NLRP3 (1:1000; Affinity Biosciences, Jiangsu province, China); p38, p-p38, ERK, p-ERK (Cell Signaling Technology, USA); myD88, JNK, p-JNK, p65, p-p65 (Proteintech Group, Wuhan, China); β-actin (ABclonal, China).

### Data analysis

Statistical analysis was performed using IBM SPSS Statistics 21 software and GraphPad Premier 6.0 software package (GraphPad Prism). The differences between the groups were statistically calculated by one-way analysis of variance (ANOVA). Data are presented as the mean ± standard error of the mean (SEM), and considered significant at ^*^ *P* < 0.05, ^**^ *P* < 0.01, ^***^ *P* < 0.001, ^****^ *P* < 0.0001; ns: non-significant.

## Results

### HcESPs promoted the polarization of PBMCs toward anti-inflammatory and immunosuppressive phenotypes

The proliferation of PBMCs incubated with various doses (10, 20, 40, and 80 μg/ml) of HcESPs was considerably restrained when compared with the control group (**Supplementary material: Figure S2**).

**Figure 1:**
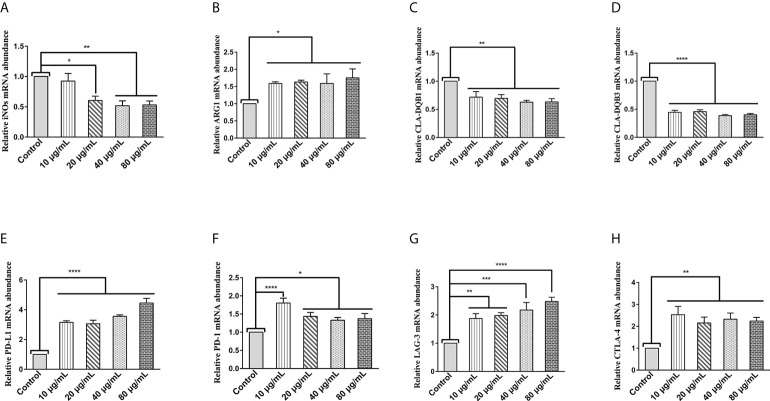
HcESPs upregulated the transcript levels of Arg1 and immune checkpoint molecules in PBMCs, while downregulated the transcript levels of iNOs and MHC II in PBMCs. Cells were incubated with different concentrations (10, 20, 40, 80 μg/mL) of HcESPs or an equal volume of RPMI 1640 medium (control) at 37°C and 5% CO_2_ for 24 h. **(A, B)** The transcript levels of iNOs and Arg1 were detected by qPCR assay; **(C, D)** The transcript levels of MHC II (CLA-DQB1 and CLA-DQB3) were detected by qPCR assay; **(E-H)** The transcript levels of immune checkpoints molecules (PD-L1, PD-1, LAG-3, CTLA-4) were detected by qPCR assay; Data are presented as the mean ± SEM from three independent experiments. * *P* < 0.05, ** *P* < 0.01, *** *P* < 0.001, **** *P* < 0.0001 vs the control group.

The transcript levels of specific phenotypic markers of M1 (inducible nitric oxide synthase (iNOS)) and M2 (arginase-1(Arg1)) macrophages were assessed by qPCR assays. As shown in **Figure 1A**, HcESPs significantly down-regulated the transcript levels of iNOs in PBMCs, while significantly up-regulating the transcript levels of Arg1 (**Figure 1B**). This suggests that HcESPs may have contributed to the polarization of macrophages toward M2 macrophages.

Extracellular peptides (e.g., bacteria and helminths) are presented to CD4+- T cells *via* major histocompatibility complex (MHC) class II molecules (29). MHC class II molecules play an essential role in fighting against parasitic infections due to their known antigen presentation function (30, 31). Therefore, we evaluated the effect of HcESPs on the transcript levels of MHC class II molecules (CLA-DQBI, CLA-DQB3) in PBMCs by qPCR assays. As shown in **Figures 1C, D**, HcESPs significantly downregulated the mRNA expression levels of antigen-presenting molecules (CLA-DQBI, CLA-DQB3) in PBMCs, suggesting that HcESPs affected the antigen-presenting function in PBMCs. In addition, HcESPs significantly inhibited the ability of PBMCs to take up FITC-dextran (**Supplementary material: Figure S3**), suggesting that HcESPs inhibited the phagocytosis in PBMCs.

LAG-3, PD-1, CTLA-4, and PD-L1 are suppressive immune checkpoints impair lymphocyte function (9, 32, 33). Similarly, the expression of these immune checkpoint molecules plays a vital role in the parasite’s evasion of the host immune system (34–38). Therefore, in this study, the effect of HcESPs on the transcript levels of suppressive immune checkpoint molecules in PBMCs was assessed by qPCR assays. Interestingly, different concentrations of HcESPs significantly upregulated the mRNA levels of PD-L1, PD-1, LAG-3, and CTLA-4 in PBMCs (**Figures 1E–H**), suggesting that HcESPs impaired T-cell function and created an “immunosuppressive microenvironment”. Interestingly, the transcriptional levels of PD-1 in the HcESPs groups of 20, 40, and 80 μg/mL showed a down-regulation trend compared with the HcESPs group of 10 μg/mL. This suggested that the transcriptional regulation of PD-1 in PBMCs was diminished when the concentration of HcESPs reached 20 μg/mL. This may be related to negative feedback regulation of PD-1 transcription in PBMCs. The precise regulatory mechanism needs to be further elucidated by subsequent studies.

### HcESPs promoted Treg cell proliferation and differentiation

**Figure 2:**
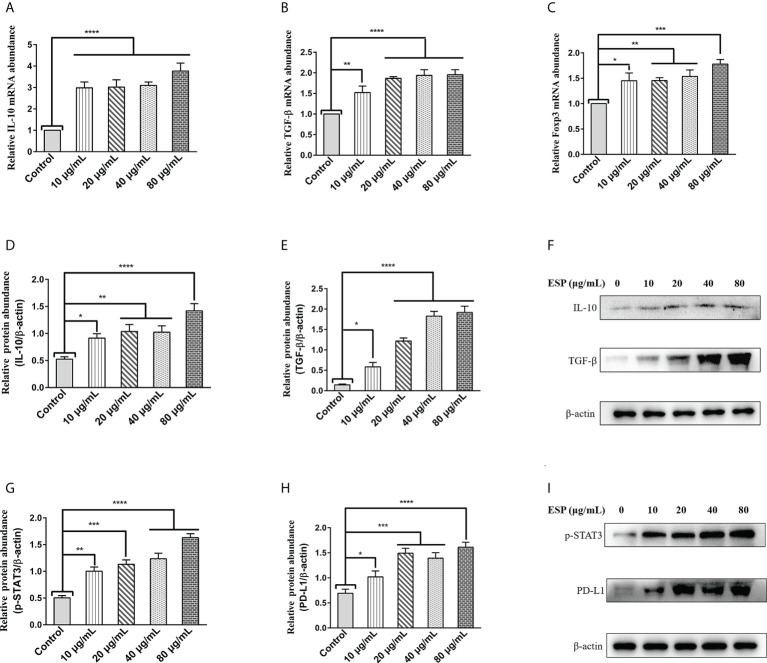
HcESPs promoted the expression of Treg cell markers and activated the STAT3/PD-L1 pathway. Cells were incubated with different concentrations (10, 20, 40, 80 μg/mL) of HcESPs, and an equal volume of RPMI 1640 medium (control) at 37°C and 5% CO_2_ for 24 h. **(A–C)**: The transcript levels of IL-10, TGF-β, and Foxp3 were detected by qPCR assays; **(D–F)**: Detection of relative protein expression levels of IL-10 and TGF-β by Western blot; **(G–I)**: Detection of relative protein expression levels of p-STAT3, PD-L1 by Western blot; Data are presented as the mean ± SEM from three independent experiments. * *P* < 0.05, ** *P* < 0.01, *** *P* < 0.001, **** *P* < 0.0001 vs the control group.

Regulatory T (Treg) cells are specialized immunosuppressive CD4+ T lymphocytes that play a critical role in parasite immune escape (39, 40). The transcription factor Forkhead box protein 3 (Foxp3) is a marker molecule of Treg cells (41, 42). The qPCR results showed that different concentrations of HcESPs treatment significantly upregulated the transcript levels of IL-10, TGF-β, and Foxp3 in PBMCs (**Figures 2A–C**). Moreover, western blot results showed that HcESPs significantly promoted the protein expression levels of IL-10 and TGF-β in PBMCs (**Figures 2D–F**). These results suggest that HcESPs promote the proliferation and differentiation of Treg cells in PBMCs.

### HcESPs activated the STAT3/PD-L1 pathway in PBMCs

A growing body of evidence suggests that STAT3 activation is a critical molecular hub for immune evasion by bacteria, tumors, etc. (43–45). However, is it possible that worm ESPs activates STAT3/PD-L1 pathway like tumor cells, bacteria, and other pathogens, which has not been reported yet? Therefore, we analyzed the effect of HcESPs treatment on the STAT3/PD-L1 pathway in PBMCs by western blot assays. Interestingly, western blot results (**Figures 2G-I**) showed that different concentrations of HcESPs treatment significantly upregulated the protein expression levels of p-STAT3, and PD-L1, suggesting that HcESPs activated the STAT3/PD-L1 pathway in PBMCs.

### HcESPs down-regulated the transcript levels of TLR2, TLR4, and TLR5 and up-regulated the transcript levels of TLR1 in PBMCs

**Figure 3:**
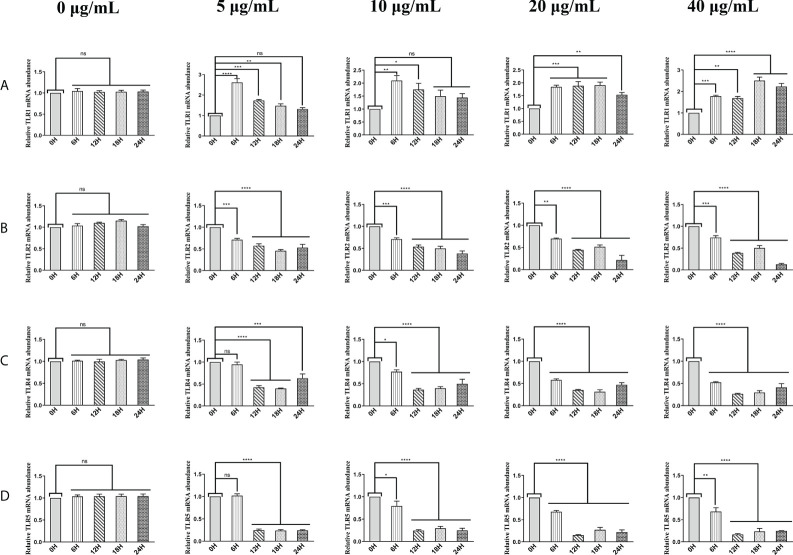
HcESPs down-regulated the transcript level of TLR2, TLR4, and TLR5 while up-regulated the transcript level of TLR1 in PBMCs. Cells were incubated with different concentrations (0, 5, 10, 20, 40 μg/mL) of HcESPs at 37°C and 5% CO_2_ for 0, 6, 12, 18, and 24 hours, respectively. **(A–D)**: The transcript levels of TLR1, TLR2, TLR4, and TLR5 were detected by qPCR assays; Data are presented as the mean ± SEM from three independent experiments. Asterisks indicate significant differences at *P < 0.05, **P < 0.01, ***P < 0.001, and ****P < 0.0001; ns: non-significant.

The above results suggest that HcESPs inhibit the adaptive immune response and create a “microenvironment” of immunosuppression. We next analyzed the effect of HcESP on PRR in PBMCs. We analyzed the changes in mRNA levels of TLR1, TLR2, TLR3, TLR4, TLR5, TLR6, TLR7, TLR8, TLR9, TLR10, C-type lectin MCL (CLEC4D), macrophage-inducible C-type lectin (CLEC4E), a significant decrease in C-terminal caspase recruitment domain [CARD] domain-containing protein 4 (NLRC4), and MDA5 by qPCR assays when different concentrations of HcESPs were co-incubated with PBMCs for different times. The results are shown in **Figure 3**. HcESPs significantly down-regulated the transcript levels of TLR2, TLR4, and TLR5 in PBMCs and showed a dose-dependent relationship (**Figures 3B–D**) while up-regulating the transcript level of TLR1 in PBMCs and showed a dose-dependent relationship (**Figure 3A**). However, the effect of HcESPs on the transcript levels of TLR3, TLR6, TLR7, TLR8, TLR9, TLR10, CLEC4D, CLEC4E, MDA5, and NLRC4 in PBMC showed turbulent changes over time such as up-regulation or down-regulation (**Supplementary material: Figure S4**).

HcESPs inhibit the expression of TLR2, TLR4, and TLR5, which may be one of the mechanisms by which HcESPs suppress the host inflammatory response. It is well known that TLR4/NF-κB is a classical pro-inflammatory pathway, and activation of the TLR4/NF-κB signaling pathway promotes the release of inflammatory mediators such as TNF-α, IL-6, and IL-1β. Therefore, we next analyzed the effect of HcESPs on TLR4-related inflammatory pathways.

### HcESPs downregulated the levels of pro-inflammatory mediators in PBMCs

**Figure 4:**
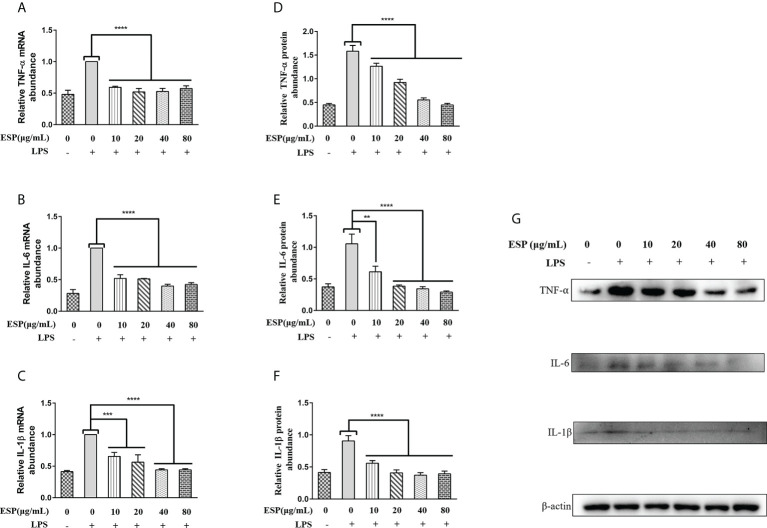
HcESPs reversed the LPS-induced upregulation of inflammatory mediators in PBMCs. Cells were stimulated with LPS (1 μg/mL) to establish an inflammation model, followed by incubation with different concentrations (0, 10, 20, 40, 80 μg/mL) of HcESPs at 37°C and 5% CO_2_ for 24 h. A blank control group was set up (equal volumes of RPMI 1640 were used instead of LPS and HcESPs). **(A–C)**: Detection of relative transcript levels of TNF-α, IL-6, and IL-1β in PBMCs by qPCR assays; **(D–G)**: Detection of relative protein expression levels of TNF-α, IL-6, and IL-1β by Western blot; Data are presented as the mean ± SEM from three independent experiments. Asterisks indicate significant differences at **P < 0.01, ***P < 0.001, and ****P < 0.0001.

As shown in **Figures 4A–C**, LPS upregulated the transcript levels of TNF-α, IL-6, and IL-1β in PBMCs. However, HcESPs treatment significantly reversed the LPS-induced upregulation of the transcript levels of pro-inflammatory mediators (TNF-α, IL-6, IL-1β) (**Figures 4A–C**). Consistent with the qPCR results, western blot results (**Figures 4D–G**) showed that HcESPs significantly reversed the upregulation of pro-inflammatory mediator (TNF-α, IL-6, IL-1β) protein expression levels caused by LPS. These results suggest that HcESPs can attenuate the inflammatory response of PBMCs due to LPS.

The mRNA level of TNF-αremains constant irrespective of the increase in the concentration of HcESP, but the relative protein abundance gradually decreases. This may indicate that the regulation of TNF-α transcription in PBMCs by HcESPs saturates when HcESPs reaches 10 µg/mL, and the transcriptional regulation of TNF-α is not enhanced with its increasing concentration. However, the regulation of post-transcriptional translation of TNF-α by HcESPs in PBMCs showed a dose-dependent relationship with increasing concentration. The precise regulatory mechanism needs to be further studied.

### HcESPs reversed LPS-induced activation of the TLR4/NF-κB signaling pathway

**Figure 5:**
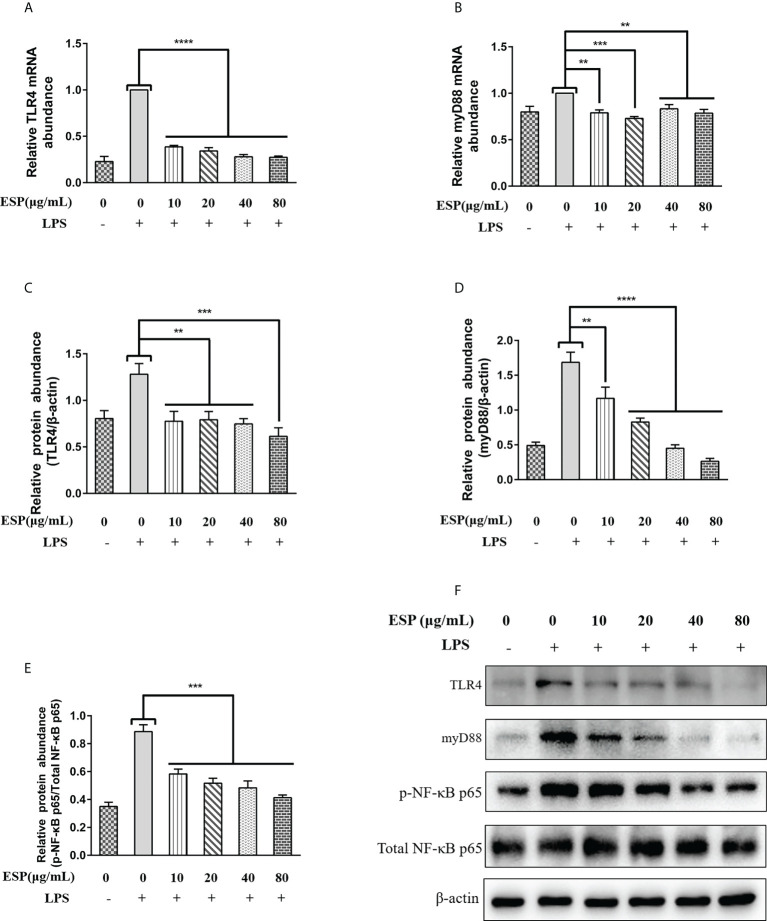
HcESPs reversed LPS-induced activation of the TLR4/NF-κB signaling pathway. Cells were stimulated with LPS (1 μg/mL) to establish an inflammation model, followed by incubation with different concentrations (0, 10, 20, 40, 80 μg/mL) of HcESPs at 37°C and 5% CO_2_ for 24 h. A blank control group was set up (equal volumes of RPMI 1640 were used instead of LPS and HcESPs). **(A, B)**: Detection of relative transcript levels of TLR4 and myD88 in PBMCs by qPCR assays; **(C–F)**: Detection of relative protein expression levels of TLR4, myD88, p65, and p-p65 by Western blot; Data are presented as the mean ± SEM from three independent experiments. Asterisks indicate significant differences at **P < 0.01, ***P < 0.001, and ****P < 0.0001.

We examined the effects of HcESPs on the transcriptional expression of key proteins of the TLR4/myD88/NF-κB signaling pathway by qPCR and western blot assays. The qPCR results (**Figures 5A, B**) showed that HcESPs significantly reversed the LPS-induced upregulation of mRNA levels of TLR4 and myD88. Consistent with the expected results, HcESPs treatment significantly down-regulated the protein expression levels of TLR4, myD88, and p-p65 in PBMCs compared with the LPS-treated group (**Figures 5C–F**). This suggests that HcESPs inhibit the TLR4/myD88/NF-κB signaling pathway in PBMCs.

The down-regulation of myD88 transcript levels showed no dose-dependent relationship with the concentration of HcESPs, while the downregulation of myD88 protein levels showed a dose-dependent relationship with the concentration of HcESPs. This suggests that the transcriptional regulation of myD88 in PBMCs may be saturated when the HcESPs reaches 10 μg/mL.

### HcESPs inhibited MAPKs signaling pathway in PBMCs

**Figure 6:**
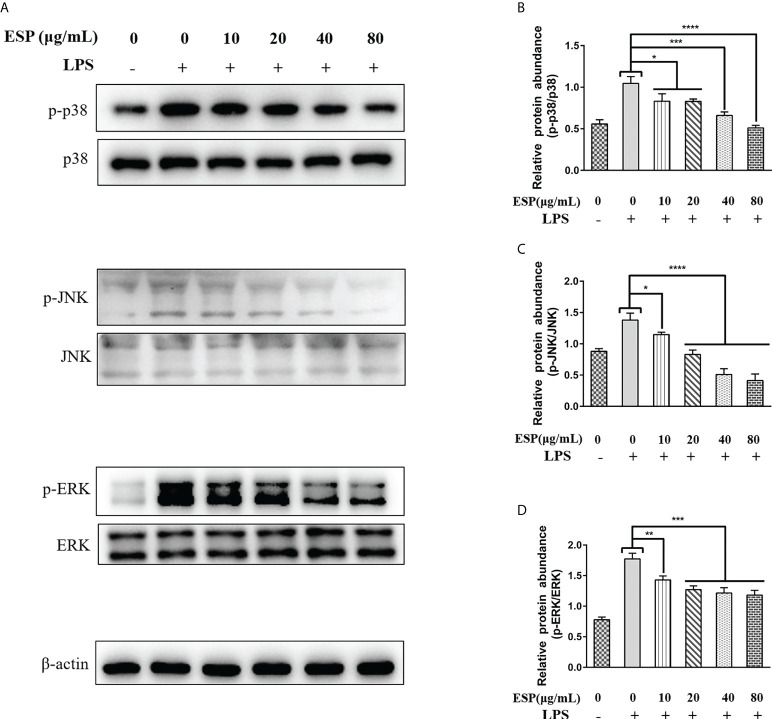
HcESPs inhibited the activation of the MAPKs signaling pathway. Cells were stimulated with LPS (1 μg/Ml) to establish an inflammation model, followed by incubation with different concentrations (10, 20, 40, 80 μg/mL) of HcESPs or equal volumes of RPMI 1640 medium at 37°C and 5% CO_2_ for 24 h. A blank control group was set up (RPMI 1640 instead of LPS and HcESPs). **(A)**: Detection of protein expression levels of p38, p-p38, JNK, p-JNK, ERK, and p-ERK by Western blot; **(B–D)**: Statistics of the p-p38, p-JNK, and p-ERK; Data are presented as the mean ± SEM from three independent experiments. Asterisks indicate significant differences at *P < 0.05, **P < 0.01, ***P < 0.001, and ****P < 0.0001.

It is well known that activation of the MAPKs signaling pathway promotes inflammatory responses, which in turn promotes the release of pro-inflammatory mediators and plays a vital role in host immune defense. Therefore, we analyzed the effect of HcESPs treatment on the expression levels of key proteins of MAPKs signaling pathway in PBMCs using western blot. Interestingly, HcESPs treatment significantly reversed the LPS-induced upregulation of p-p38, p-JNK, and p-ERK protein phosphorylation levels in PBMCs (**Figures 6A–D**), indicating that HcESPs ameliorated LPS-induced activation of the MAPKs signaling pathway.

### HcESPs inhibited the NLRP3 signaling pathway in PBMCs

**Figure 7:**
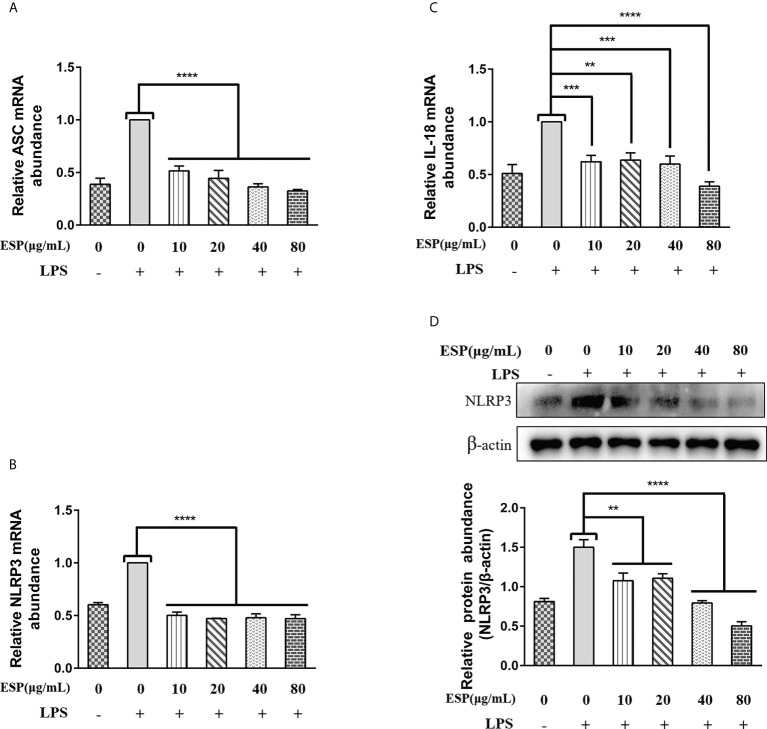
HcESPs inhibited the NLRP3 signaling pathway in PBMCs. Cells were stimulated with LPS (1 μg/mL) to establish an inflammation model, and incubated with different concentrations (10, 20, 40, 80 μg/mL) of HcESPs or equal volume of RPMI 1640 medium at 37°C and 5% CO_2_ for 24 h. A blank control group was set up (equal volume of RPMI 1640 instead of LPS and HcESPs). **(A–C)**: Detection of relative transcript levels of ASC, NLRP3, and IL-18 in PBMCs by qPCR assays; **(D)**: Detection of relative protein expression of NLRP3 by Western blot; Data are presented as the mean ± SEM from three independent experiments. Asterisks indicate significant differences at **P < 0.01, ***P < 0.001, and ****P < 0.0001.

Activation of the Nod-like receptor pyrin domain containing protein-3 (NLRP3) signaling pathway contributes to the release of pro-inflammatory factors that help the host resist pathogen invasion and play a key role in innate immunity. However, little is known about whether HcESPs regulate the NLRP3 signaling pathway in PBMCs. Therefore, we next analyzed the mRNA levels of ASC, NLRP3, and IL-18 in PBMCs by qPCR assays, and the protein levels of NLRP3 were detected by western blot assays. The results (**Figures 7A–C**) showed that HcESPs significantly downregulated the transcript levels of ASC, NLRP3, and IL-18 in PBMCs compared with the LPS-treated group. Consistent with the qPCR results, HcESPs significantly down-regulated the protein expression level of NLRP3 in PBMCs compared with the LPS-treated group (**Figure 7D**). This suggests that HcESPs inhibit the activation of the NLRP3 signaling pathway in PBMCs.

## Discussion

Many studies have reported that the ESPs of worms play a crucial role in aiding worm infection and parasitism (46–48). Previous studies in our laboratory have shown that a single component protein from HcESPs has a significant protective effect as a vaccine candidate (49–52). This may be because the antibodies blocked the immunosuppressive molecules in HcESPs, which reactivated the host immune function. Interestingly, passive immunization with IgG against a single component protein from HcESPs also achieved a significant protective effect against *H. contortus* infection (anti-*H. contortus* α/β-hydrolase domain protein IgG: 54% reduction in fecal egg counts and 74.2% reduction in worm burden; anti-*H. contortus* adhesion-regulating molecule 1 IgG: 48.9% reduction in fecal egg counts and 58.6% reduction in worm burden; anti-*H. contortus* transthyretin domain–containing protein IgG: 63.7% reduction in fecal egg counts and 66.4% reduction in worm burden) (49, 50, 52).

In adaptive immunity, antigen-presenting cells (APC) present extracellular pathogen antigen molecules to CD4-T cells *via* MHC-II molecules (53). Thus, the peptide encoded by the MHC gene constitutes the first line of defense of the host against pathogens. In the present study, we found that HcESPs significantly downregulated the mRNA levels of MHC-II (CLA-DQB1, CLA-DQB3) in PBMCs. The release of ESPs during nematode infection resulted in a decrease in the antigen presentation efficiency of APC, which may be one of the factors limiting vaccine efficacy. A growing number of studies have shown that APC-expressed MHC molecules are one of the key factors influencing immunotherapy (54–56).

Macrophages are essential in the immune response and can be divided into two types: classically activated macrophages with pro-inflammatory potency (M1, mainly expressing iNOs as a marker) and alternatively activated macrophages with anti-inflammatory function (M2, mainly expressing Arg1 as a marker) (57, 58). It has been shown that the anti-inflammatory microenvironment created by M2 macrophages contributes to tumor and parasite immune evasion (59–62). In the present study, we found that HcESPs significantly promoted the transcription of Arg1 in PBMCs and inhibited the transcription of iNOs in PBMCs, which may be the result of HcESPs promoting the polarization of macrophages to M2 macrophages in PBMCs.

Immune checkpoint blockade therapy is effective in oncology, and chronic viral infections. Development of inhibitors targeting PD-L1, PD-1, CTLA-4, and LAG-3 showed great potentialities in oncology immunotherapy (9, 63–65). In our study, results showed that HcESPs significantly upregulated the transcriptional levels of PD-L1, PD-1, CTLA-4, and LAG-3 in PBMCs and activated the STAT3/PD-L1 signaling pathway, which may be one of the mechanisms by which HcESPs promote immune evasion in *H. contortus*. Activation of immune checkpoint molecules suppressed T cell function, which may explain the downregulation of PBMCs activity due to HcESPs (7). Therefore, in subsequent studies, it is necessary to investigate the efficacy of targeted blocked immune checkpoint molecular therapies for *H. contortus* infection. The helminth parasite fights immune rejection through complex evasion mechanisms, including activation of host immunosuppressive Treg cells (66). Studies showed that Treg cells suppressed inflammatory reactions by secreting IL-10 and TGF-β, and contributed to parasite immune evasion (47, 67). In our study, we found that *H. contortus* ESPs activated goat Treg cells and promoted the secretion of IL-10 and TGF-β, which was similar with previous studies (47, 67).

The PRR-related signaling pathway is a critical link between innate and adaptive immunity and is crucial for worm invasion, survival, pathogenesis, and elimination (68, 69). TLRs can be expressed on the surface of various immune cells, such as lymphocytes, macrophages, dendritic cells, and eosinophils (70, 71). During helminth infection, various glycolipids and glycoproteins from helminths can bind to TLRs on the surface of immune cells, activating or modulating TLRs-mediated immune responses (72, 73). ESPs play an essential role in worm parasitism by interfering with TLRs-mediated immune responses. It was demonstrated that ESPs from *Trichinella spiralis* down-regulated TLR2\TLR4 mRNA expression in macrophages, suppressing the pro-inflammatory response induced by LPS (74). Recent studies have shown that ESPs of *Echinococcus granulosus* downregulated TLR2/TLR4 expression in goat PBMCs (75). Interestingly, we found that different concentrations of HcESPs co-incubated with PBMCs at different times all significantly down-regulated the mRNA levels of TLR2, TLR4, and TLR5, while up-regulating the mRNA of TLR1. Similar to the previous findings (74, 75), HcESP down-regulated TLR2 and TLR4 mRNA expression in PBMCs, which may be one of the mechanisms by which HcESPs suppress host inflammatory responses.

During helminth infection, TLR4 activation induced eosinophil infiltration and increased secretion of mucosal secretory immunoglobulin A (SIgA) antibodies in the infected area, facilitating worm expulsion (76). NF-κB is one of the core downstream signals of TLRs/MyD88, and its activation promotes the release of inflammatory mediators (TNF-α, IL-6, IL-1β) (77). Interestingly, our study found that HcESPs reversed the LPS-induced upregulation of the expression levels of inflammatory mediators. This suggests that HcESPs might have anti-inflammatory effects. We hypothesized that the downregulation of pro-inflammatory mediators in PBMCs by HcESPs might be related to the suppression of the TLR4/myD88/NF-κB signaling pathway. Consistent with the predicted results, HcESPs inhibited the expression of TLR4, myD88, and p-NF-κB P65, suggesting that HcESPs downregulate LPS-induced inflammatory responses by suppressing the TLR4/myD88/NF-κB signaling pathway.

NF-κB/MAPKs/NLRP3 is a classical signaling pathway of inflammation that regulates the release of pro-inflammatory mediators and plays a vital role in fighting against pathogen invasion. Key molecules in the NF-κB, MAPKs, and NLRP3 signaling pathways are essential targets for anti-inflammatory drug research (78–80). The activation of the p38 MAPK signaling pathway was found to promote the phosphorylation and degradation of IκBα, which activates the NF-κB signaling pathway (81). In the present study, we found that HcESPs inhibited LPS-induced activation of MAPKs (JNK, ERK, p38) signaling pathway and had the best inhibitory effect on the JNK pathway. In addition, HcESPs also inhibited the LPS-induced activation of the NLRP3 signaling pathway. These results are consistent with the downregulation of inflammatory mediator expression levels. Inhibition of TLR4/MAPKs/NF-κB/NLRP3 signaling pathway by HcESPs may be a strategy for *H. contortus* evasion of the host innate immunity.

In this study, we analyzed the mechanisms involved in suppressing the host immune system by HcESPs. However, due to limitations in the availability of goat antibodies, we only analyzed the transcriptional level of iNOs, Arg1, immune checkpoint molecules (PD-1, LAG-3, CTLA-4), MHC-II, Foxp3, pattern recognition receptors, ASC and IL-18. The changes in protein levels deserve further study.

## Conclusion

**Figure 8:**
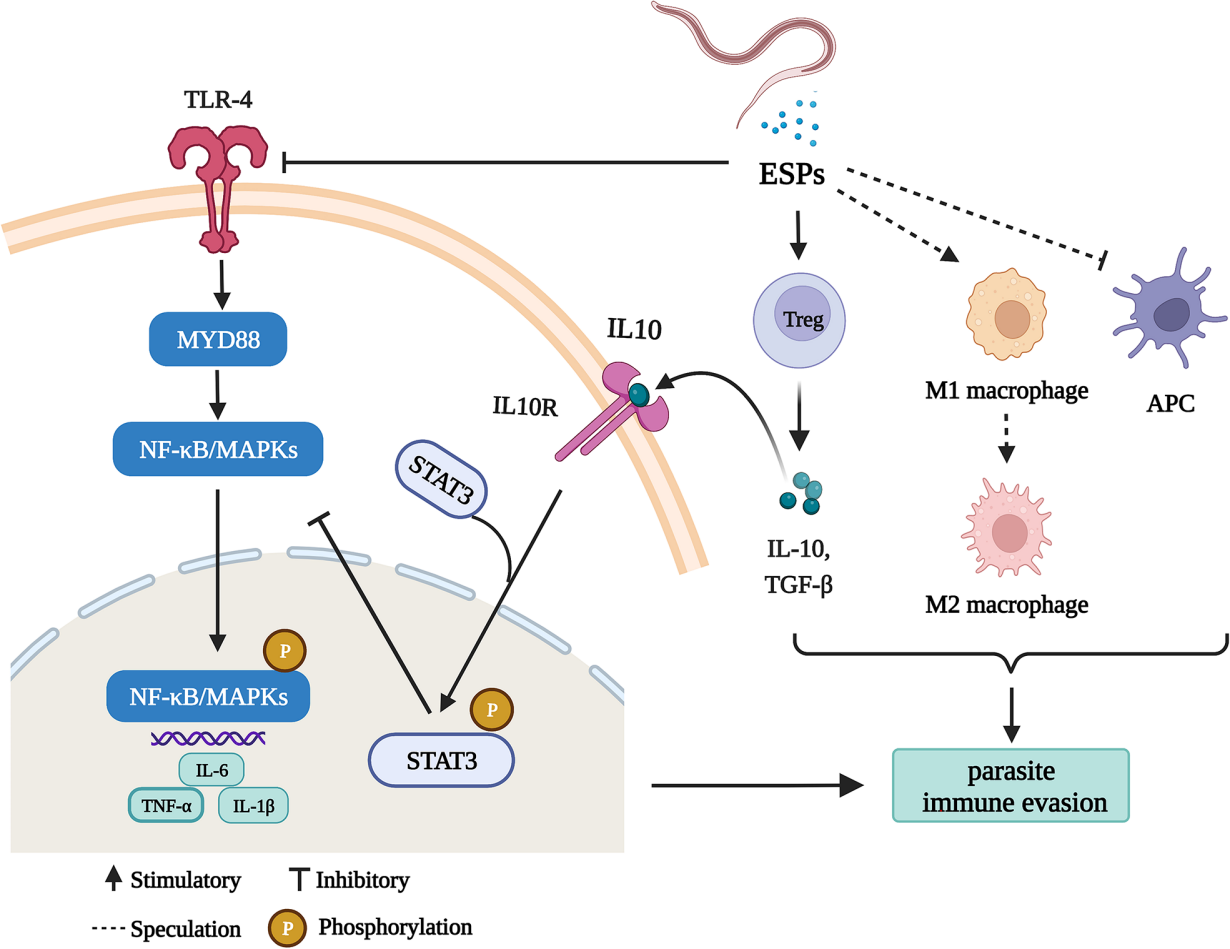
HcESPs suppress goat immune function: a possible mechanism of immune evasion in *Haemonchus contortus* (Charting with BioRender.com software). HcESPs suppress adaptive immunity by upregualtion of Treg cells, inhibiting the antigen presenting cells, and promoting the shift of macrophages from pro-inflammatory type (M1) to anti-inflammatory type (M2). In addition, HcESP suppresses innate immunity by inhibiting TLR4//NF-κB pathway and activating IL-10/STAT3 pathway, which result in downregulated inflammatory responses.

In the present study, we demonstrate several ways that HcESPs help the parasite to escape the host immunity (**Figure 8**), including (1) inhibition of antigen presentation, (2) upregulation of immune checkpoint molecules, (3) promotion of Tregs cell proliferation, (4) activation of STAT3/PD-L1 pathway, (5) down-regulated the expressions of TLR2, TLR4, and TLR5 and (6) downregulated the expression of pro-inflammatory cytokines by inhibiting the TLR4/NF-κB/MAPKs/NLRP3 signaling pathway.

## Data availability statement

The original contributions presented in the study are included in the article/**Supplementary Material**. Further inquiries can be directed to the corresponding author.

## Ethics statement

The animal study was reviewed and approved by the Science and Technology Agency of Jiangsu Province. The approval ID is SYXK (SU) 2010-0005.

## Authors contributions

Data curation, ZW and YZ. Formal analysis, ZW, JF, YZ, and KA. Funding acquisition, RY. Investigation, JF. Methodology, ZW and KA. Project administration, RY. Resources, RY. Software, ZW and KA. Supervision, ML, XS, LX, XL, and RY. Visualization, ZW and MA. Writing – original draft, ZW. Writing – review and editing, ZW and RY. All authors contributed to the article and approved the submitted version.

## Funding

This research was funded by the National Natural Science Foundation of China (31872464) and State Key Laboratory of Pathogenesis, Prevention and Treatment of High Incidence Diseases in Central Asia Fund (SKL-HIDCA-2022-20).

## Conflict of interest

The authors declare that the research was conducted in the absence of any commercial or financial relationships that could be construed as a potential conflict of interest.

## Publisher’s note

All claims expressed in this article are solely those of the authors and do not necessarily represent those of their affiliated organizations, or those of the publisher, the editors and the reviewers. Any product that may be evaluated in this article, or claim that may be made by its manufacturer, is not guaranteed or endorsed by the publisher.
