# Supplementary material for: Excretory/secretory proteins inhibit host immune responses by downregulating the TLR4/NF-κB/MAPKs signaling pathway: A possible mechanism of immune evasion in parasitic nematode Haemonchus contortus
Source: Front Immunol. 2022 Sep 27;13:1013159. doi: 10.3389/fimmu.2022.1013159 (PMC9551057; doi:10.3389/fimmu.2022.1013159)
Supplement: Supplementary file 1 [file DataSheet_1.docx]

**Fig S1**


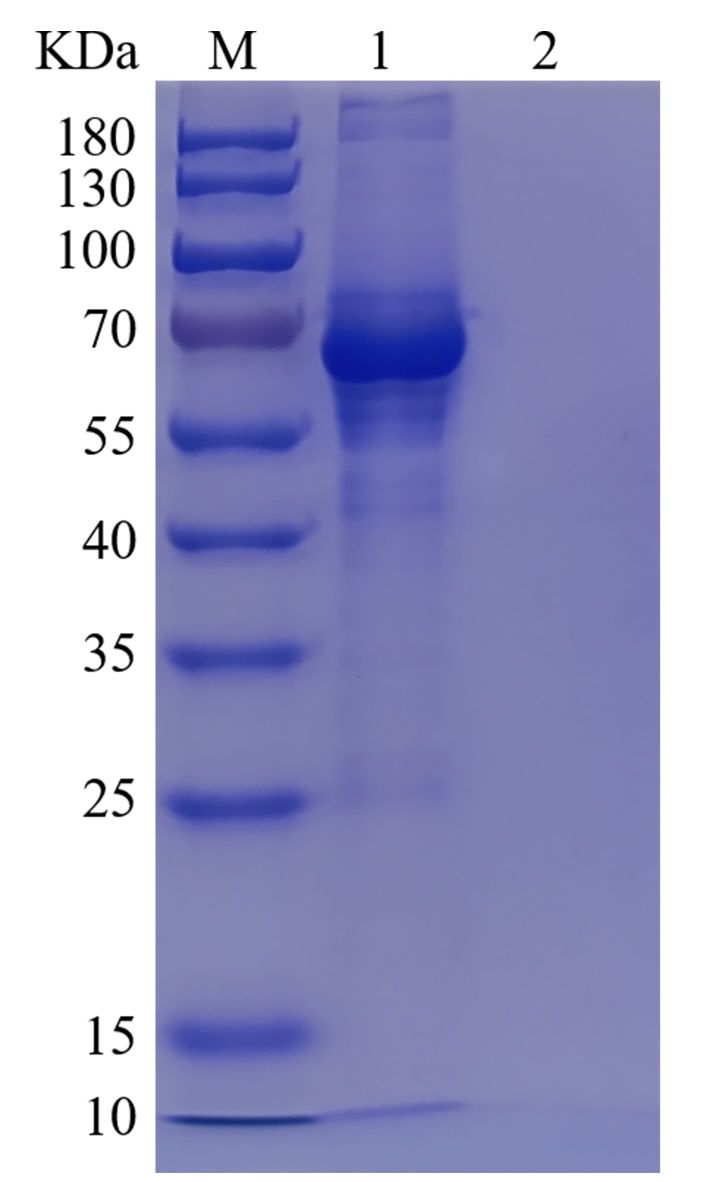


**Figure S1:** Collection of HcESPs. Lane M: standard protein molecular weight Marker; Lane 1: SDS–PAGE showing HcESP production. Lane 2: RPMI 1640 medium control. The HcESPs collection trials were derived from at least three independent replicate trials.

**Fig S2**





**Figure S2:** Effect of HcESPs on PBMC proliferation. Cells were incubated with different concentrations (10, 20, 40, 80 μg/mL) of HcESPs or with an equal volume of RPMI 1640 medium (control) at 37 °C, and 5% CO2 for 24 h.The cell proliferation index was determined by setting the OD_450_ values of the control group as 100%.Data are presented as the mean ± SEM from three independent experiments. *** *P* < 0.001, **** *P* < 0.0001 vs the control group.

**Fig S3**


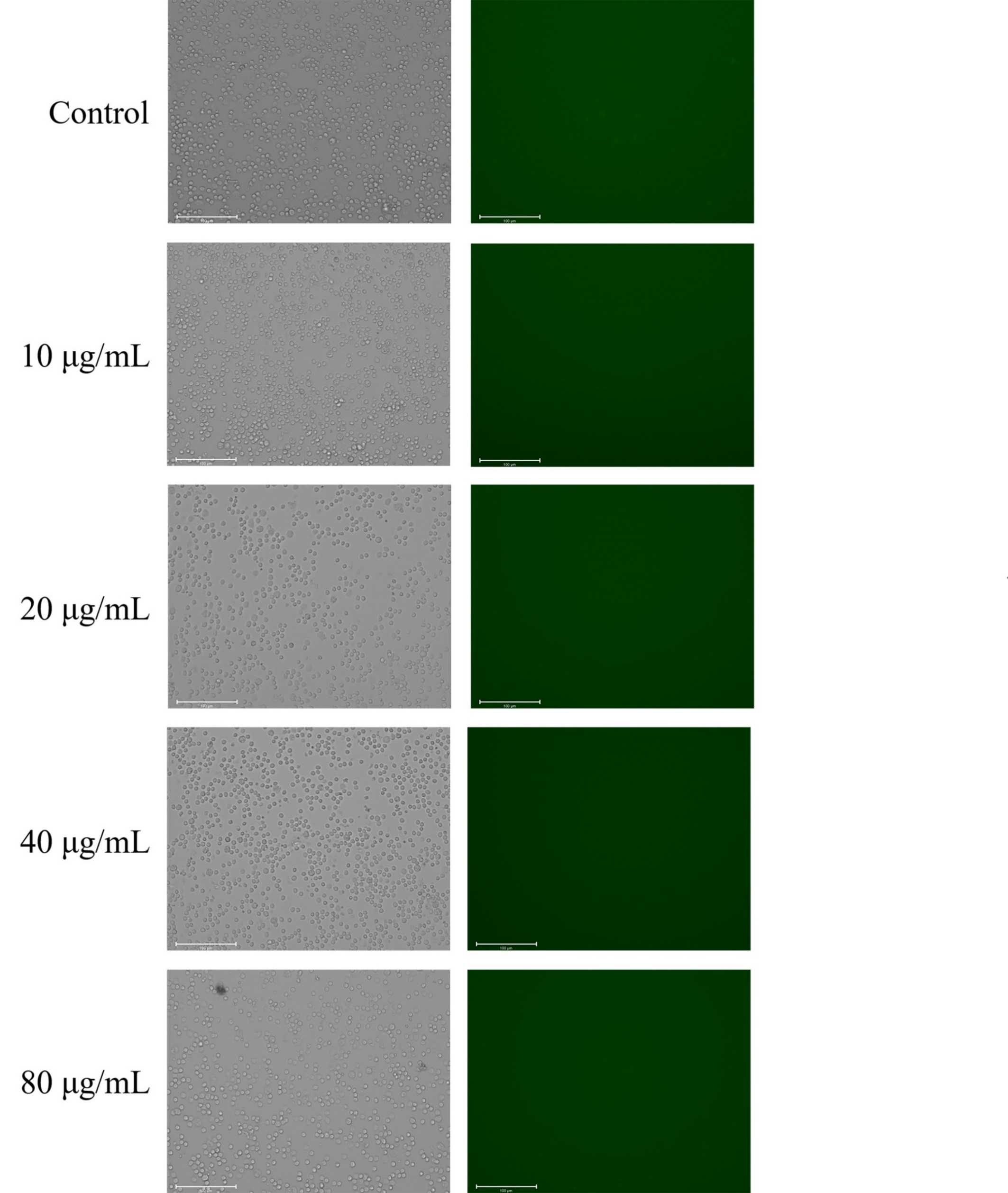


**Figure S3:** HcESPs inhibited the phagocytosis of immune cells.Cells were incubated with different concentrations (10, 20, 40, 80 μg/mL) of HcESPs, and an equal volume of RPMI 1640 medium (control) at 37 °C and 5% CO_2_ for 24 h and then incubated for 1 h with FITC-dextran (concentration: 100 ng/mL). The uptake of FITC-dextran by immune cells was observed by fluorescence microscopy to reflect the strength of phagocytosis of PBMCs. FITC-dextran was used as a fluorescent antigen, and FITC fluorescence was observed in PBMCs after phagocytosis of FITC-dextran. Therefore, the phagocytic function of PBMCs can be assessed by the presence or absence, intensity, or weakness of fluorescence. The fluorescence intensity was significantly reduced in the group treated with different concentrations of HcESPs compared with the control group. The data presented are the results of three independent experiments. Scale bars: 100 μm.

**Fig S4**

**
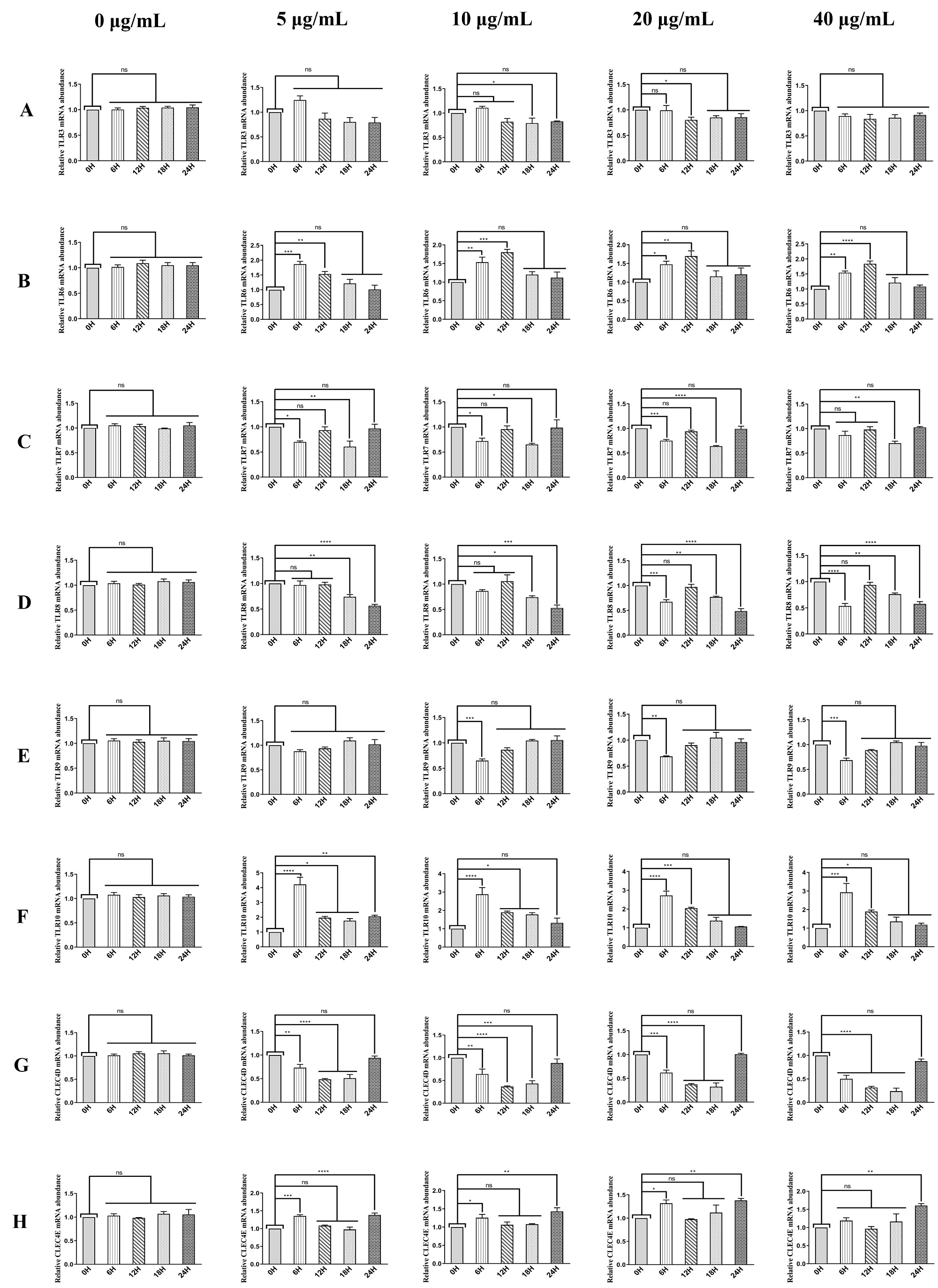
**

**
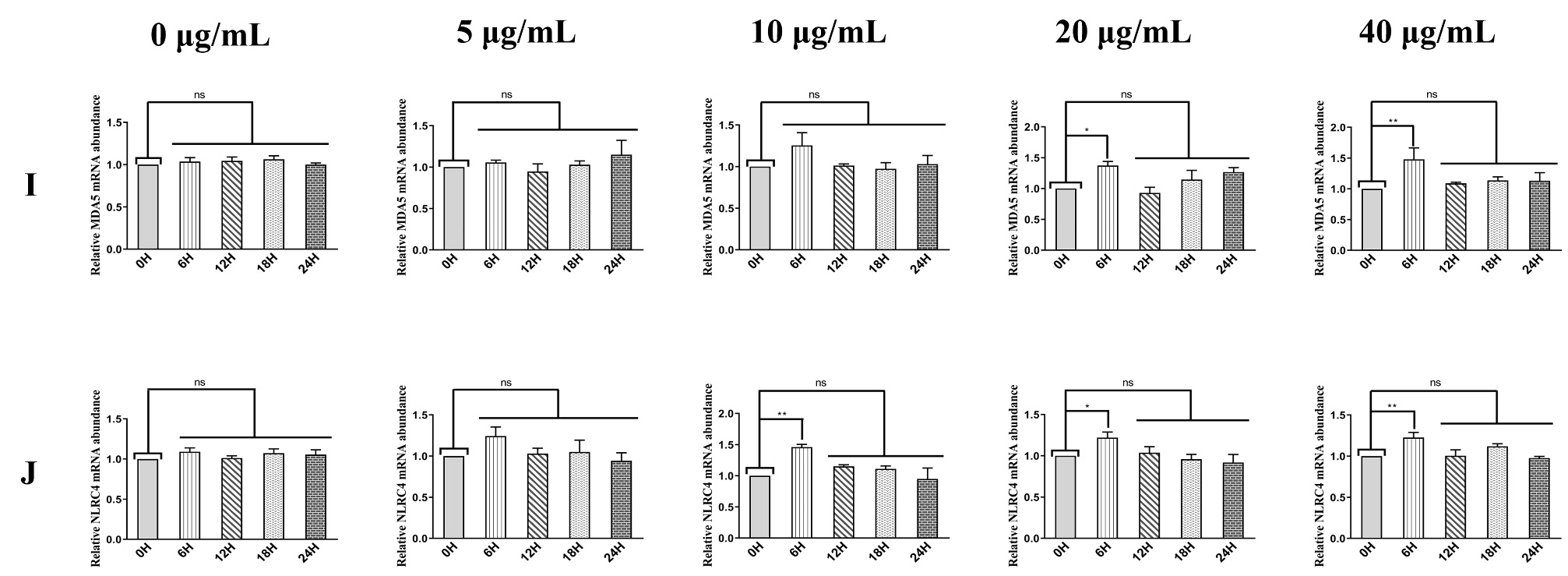
**

**Figure S4:**Effect of HcESPson the transcriptional levels of pattern recognition receptors (PRRs) in PBMCs.Cells were incubated with different concentrations (5, 10, 20, 40μg/mL) of HcESPsorequal volumes of RPMI 1640 medium (control, 0μg/mL) at 37°C and 5% CO_2_ for 0, 6, 12, 18, and 24 hours, respectively.**A-J:**The transcript levels of TLR3 (**A**), TLR6 (**B**), TLR7 (**C**), TLR8 (**D**), TLR9 (**E**), TLR10 (**F**), CLEC4D (**G**), CLEC4E (**H**), MDA5 (**I**), and NLRC4 (**J**) were detected by qPCR assays. Data are presented as the mean ± SEM from three independent experiments. Asterisks indicate significant differences at **P* < 0.05, ***P*< 0.01, ****P* < 0.001, and *****P* < 0.0001; ns: non-significant.
