# Supplementary material for: Excretory/secretory proteins inhibit host immune responses by downregulating the TLR4/NF-κB/MAPKs signaling pathway: A possible mechanism of immune evasion in parasitic nematode Haemonchus contortus
Source: Front Immunol. 2022 Sep 27;13:1013159. doi: 10.3389/fimmu.2022.1013159 (PMC9551057; doi:10.3389/fimmu.2022.1013159)
Supplement: Supplementary file 2 [file Table_1.docx]

**Table S1. Primers used for qPCR experiments**

| **Target genes** | **Primer sequences (5’-3’)** |
| --- | --- |
| β-actin | CACCACACCTTCTACAAC |
|  | TCTGGGTCATCTTCTCAC |
| CLA-DQB1  CLA-DQB3 | GGTGCGGAGTGTGAACAGATACAT |
|  | GCGGCTTCCACCTGGTAGTT  GAGTACGTGCGCTTCGACA  CACTGAAACTCTCAAAGACCCC |
| iNOs | AACGTCAGAAAAGGCCACCA |
|  | GTCTTTACCGTCCCCGTGAG |
| ARG1 | ACTGAGAAAGGCTGGTCTGC |
|  | TCTTCTTGACTTCCGCCACC |
| PD-L1 | AGTCAATGCTCCATACCGCA |
|  | TGATGCTGGTTTTGCCACTC |
| PD-1  LAG3  CTLA-4  IL-10  TGF-β  Foxp3  TNF-α  IL-6  IL-1β  NLRP3  ASC  IL-18  MyD88  TLR1  TLR2  TLR3  TLR4  TLR5 | CGGCGTCACAAGCTTCATTC |
|  | GCATACTCTGTCTGCTCGGG  GAAAACCCTTCTGTGCGGTG  CACCTGGATGAGACAGCTCG  GGCAGCAGTTAGTTCAGGGT  GCTCTGTTGGGGGCATTTTC  CCTTGTCGGAAATGATCCAG  AGGGCAGAAAACGATGACAG  GAACTGCTGTGTTCGTCAGC  TCCAGGCTCCAGATGTAAGG  AAGAATGCCATCCGCCACAACC  CTCAAACTCATCCACGGTCCACAC  CCCTTGTTCCTCACCCACAC  CTCCCTGGTAGATGGGTTCG  CGTCGACAAAATCTCTGCAA  TTCCCTCAAACTCGTTCTGG  CTGTCTTGTAGAAGACGAATCG  CCGTGATGATGACCTGAGGAG  CCGTCTGGGTGAGAGCGTGAA  TCCTGTTGGCTCCTGTGTTCCT  GCCGTGGACCTTACCGACAA  GCAGTCCTGGCTTGGCTATCTT  ACTGTTCAGATAATGCACCCCAG  TTCTTACACTGCACAGAGATGGTTAC  ACAAGCCAATGAAGAAAGAG  GAGGCGAGTCCAGAACC  CTTGCCACCCTACTCTGAACC  CTAAGACCAATAAAACCTTGCTGA  CTCCCACTTCCGTCTCTTTGAT  CTCCAGGTAGGTCCTGGTGTTC  ACAACTTAGCTCGGCTCTGGA  CCTTGAAGGCTTCCACTGGGA  TTCAACCGTATCACGGCCTC  TGACCCACTGCAGGAAACTC  CCTATTGTATGTGAGCAGGAGTT  TGTGATGGGTAAGAACCAAAGAG |

| **Target genes** | **Primer sequences (5’-3’)** |
| --- | --- |
| TLR6 | TCCAAAGTGTAGGCCAAGTATCAA |
|  | AATCAGCAGAGCTGTGTTGCA |
| TLR7  TLR8  TLR9  TLR10  NLRC4  CLEC4D  CLEC4E  MDA5 | TTGAGAAGCCCCTTCAGAAGTC  TCAGACACTGCCAGAAGTACGG |
|  | AGAGGCTAATGGAGGAGA  TCGTTGGCTGTTAGGAC  AATCTCCAACCGCATCCACC  AGCGACAGGGATACGAGAGA  TGCCTCATTTGAAAACTCTC  CAAGCACCTGAAAACAGAAT  AAGGGGAATCAGGCAAAGGG  CCCTGCTCAGACGAAGAAAGA  GGCATTGGGTGGACAAGAAAC  GGAAAATCACTCCAGGCCCA  TCCACATCACCAGCATCACAA  TAAAACAGGCACTCAGGAGCA  AACACCTTGGAGAGGGGGAA  AGAAGGTTCAGCAGTTGGGG |
